# Performance analysis of DFIG support microgrid using GA optimized restricted Boltzmann Machine algorithm

**DOI:** 10.1016/j.heliyon.2024.e30669

**Published:** 2024-05-07

**Authors:** Rajeswari Bhol, Sarat Chandra Swain, Ritesh Dash, K. Jyotheeswara Reddy, C. Dhanamjayulu, Hossam Kotb, Ahmed Emara

**Affiliations:** a School of Electrical Engineering, KIIT Deemed to be University, Bhubaneswar, India; b School of Electrical and Electronics Engineering, REVA University, Bengaluru, India; c School of Electrical Engineering, Vellore Institute of Technology, Vellore, India; d Department of Electrical Power and Machines, Faculty of Engineering, Alexandria University, Alexandria 21544, Egypt; e Electrical Engineering Department, University of Business and Technology, Ar Rawdah, Jeddah, 23435, Saudi Arabia

**Keywords:** Algorithm, GA, PSO, PSO-LSTM, Search space, Boltzmann Machine Algorithm

## Abstract

Voltage and reactive power regulation in a deregulated microgrid can be achieved by strategically placing the Static Synchronous Compensator (STATCOM) in coordination with other renewable energy sources, thus ensuring high-end stability and independent control. STATCOM plays a crucial role in effectively addressing power quality issues such as voltage fluctuation and reactive power imbalances caused by the intermittent nature of wind energy conversion systems. To successfully integrate STATCOM into the existing system, it is essential that the control system employed for STATCOM coordination aligns with the Doubly-Fed Induction Generator (DFIG) controller within the microgrid. Therefore, an efficient control algorithm is required in the microgrid, capable of coordinating with the DFIG controller while maintaining system stability. The utilization of a Genetic Algorithm (GA) in calibrating the Restricted Boltzmannn Machine (RBM) can streamline the process of determining optimal hyperparameters for specific tasks, eliminating the need for computationally intensive and time-consuming grid searches or manual tuning. This approach is particularly advantageous when dealing with large datasets within short time durations. In this research, a Simulink model comprising a DFIG-based microgrid and STATCOM has been developed to demonstrate the effectiveness of the proposed control system using RBM in managing STATCOM and facilitating microgrid operations.

## 1 Introduction

The utilization of wind energy in microgrids has gained prominence in recent years due to its potential to decrease dependence on traditional energy sources and mitigate carbon emissions. Numerous countries worldwide have been investing in wind microgrid systems. For example, the Wind Energy Technologies Office of the Department of Energy in the United States has been actively promoting the development of wind energy systems in microgrids to improve energy reliability and resiliency. In Europe, countries such as Denmark, Germany, and Spain have been at the forefront of the development and adoption of wind energy systems in microgrids [1], [2]. Moreover, countries in Asia, including China and India, have been increasing their investments in wind energy and microgrid technologies. The projection of the International Energy Agency (IEA) in a recent report suggests a significant rise in wind power generation in microgrids in the future. As per the report, the capacity of wind power generation in microgrids is expected to increase to 9.2 GW by 2024, from 3.6 GW capacity in 2019 [3], [4]. Furthermore, the report emphasizes that microgrids, including those powered by wind energy, will be more widespread in remote regions and islands, as well as in urban areas with high electricity consumption [5], [6].

The article [7], centers on the use of a Static Synchronous Compensator (STATCOM) as a solution to improve power quality in wind energy conversion systems connected to microgrids. The paper suggests a control strategy that utilizes a fuzzy logic controller to regulate the STATCOM and minimize voltage fluctuations while enhancing power quality within the microgrid. By regulating the reactive power of the STATCOM, the control strategy aims to maintain the voltage of the microgrid within acceptable limits. The study uses simulation results to illustrate the efficacy of the proposed control strategy in reducing voltage fluctuations and enhancing power quality in the microgrid. The study highlights the STATCOM's potential to enhance power quality and maintain voltage stability in wind energy conversion systems connected to microgrids [8].

The article [9], [10] proposes a new approach for the optimal placement and capacity determination of a wind-STATCOM in a microgrid. The study employs a hybrid optimization algorithm that combines particle swarm optimization (PSO) and gravitational search algorithm (GSA) to determine the best location and size of the wind-STATCOM in the microgrid. The proposed approach aims to minimize power losses, improve voltage profile, and reduce the total cost of the system by considering several factors such as the wind turbine location, wind-STATCOM capacity, and microgrid operating conditions. The effectiveness of the proposed approach is demonstrated through simulation results, which show a considerable reduction in power losses, enhanced voltage stability, and optimal siting and sizing of the wind-STATCOM in the microgrid [11], [12]. The study highlights the potential of the hybrid optimization algorithm to improve the efficiency and performance of microgrids in wind energy conversion systems.

The paper [13], [14] presents a control strategy for a wind-STATCOM in a microgrid to improve power quality and voltage stability. The study proposes the use of a proportional-integral (PI) controller to regulate the STATCOM's reactive power and improve the microgrid's voltage profile. Additionally, a fuzzy logic controller (FLC) is used to manage the power fluctuations of the wind turbine and maintain the stability of the microgrid. Simulation results demonstrate the effectiveness of the proposed control strategy in enhancing power quality and voltage stability in the microgrid. The paper [15] proposes an approach for optimal power flow in a wind-STATCOM microgrid by using a genetic algorithm to minimize generation costs and improve voltage stability. The proposed approach takes into account the variability of renewable energy sources and load forecast to ensure optimal operation of the microgrid.

The paper [16] presents an analysis of two voltage control methods for wind energy systems: Wind-STATCOM and Wind-STATCOM. The study compares the performance of both methods in terms of voltage regulation, power factor improvement, and power quality enhancement in a microgrid. The findings of the study suggest that both Wind-STATCOM and Wind-STATCOM methods are effective in enhancing voltage stability and decreasing power losses in the microgrid. However, the study concludes that Wind-STATCOM outperforms Wind-STATCOM in terms of voltage regulation and power quality improvement.

The paper [17], [18] proposes a new control strategy for the STATCOM-Wind system in microgrids using support vector regression (SVR) to predict wind power output and adjust the STATCOM output voltage. The aim of this approach is to improve the voltage stability and power quality of the microgrid.

To maintain a stable voltage level in a microgrid with STATCOM and wind turbine control, recurrent neural networks (RNNs) can be utilized to predict wind turbine output and adjust STATCOM output voltage. By analyzing wind speed, power output, and voltage data, RNNs can identify correlations and predict future wind power output, enabling STATCOM control. RNNs have the advantage of managing non-linear relationships between variables and handling long-term dependencies. Furthermore, they can adjust to changing conditions and update their predictions in real-time, making them ideal for dynamic systems like microgrids. One limitation of RNNs is the possibility of encountering the vanishing gradient problem, where the gradients become too small during backpropagation, leading to difficulty in learning long-term dependencies [19].

Restricted Boltzmannn Machines (RBMs) have been applied in the field of STATCOM to anticipate the voltage response of microgrids to changes in load or renewable energy sources. RBMs are a type of neural network that can learn the underlying distribution of input data by modeling the joint probability distribution between inputs and outputs. RBMs offer potential benefits in STATCOM control, including their ability to handle complex, non-linear relationships between input and output variables. Furthermore, RBMs can adapt to changing conditions and update their predictions in real time, making them suitable for dynamic systems like microgrids [20], [21].

Table-1 represents the performance matrices comparison of Genetic Algorithm (GA) and Particle Swam Optimization (PSO) for different electrical parameters. As observed from the Table 1, the first comparison includes the power system stability, where frequency stability of GA and PSO is marginally stable and in voltage stability, Voltage Recovery Time (VRT) is derived from IEEE Standard 1547 for both GA and PSO. Islanding stability for GA is marginally stable whereas for PSO it is unstable. The second comparison in performance matrices is done for the power quality of GA and PSO. As noted the power fluctuation of GA is 12.6% in 17 minutes of interval and for PSO the power fluctuation is of 14.01% in 20 minutes interval. Furthermore, the voltage and current values during harmonic distortion in GA will be 7.41% and 12.23%. Similarly, for PSO the voltage and current values during harmonic distortion will be 6.99% and 9.64% respectively.

**Table 1:** Performance Comparison Matrices between GA and PSO based on Power System Analysis.

Power System Stability				

Algorithm Type	Frequency Stability	Voltage Stability	Islanding stability	

GA [22]	Marginal Stable	VRT= IEEE Std 1547	Marginal Stable	
PSO [23]	Marginal Stable	VRT= IEEE Std 1547	Unstable	

Power Quality				

	Power Fluctuations	Harmonic Distortion		

GA [22], [24]	12.6% in 17 min Interval	Voltage-7.41% & Current- 12.23%		
PSO [25]	14.01% in 20 min Interval	Voltage-6.99% & Current- 9.64%		

Control Performance				

	Response time (ms.)	Overshoot (ms.)	Settling time (ms.)	

GA [26]	87	1.71	11.07	
PSO [27]	63	1.96	11.44	

Power Loss				

	Energy loss	Power loss percentage		

GA [27], [28]	14.21%	21.00%		
PSO [29]	17.49%	18.87%		

As observed the fourth comparison in Table 1 of performance matrices is of power loss for GA and PSO. The energy loss of GA is 14.21% and for PSO it is 17.49% whereas the power loss percentage of GA is 21.00% and 18.87% respectively. Control performance in performance matrices for GA and PSO is the last comparison in the Table 1, where response time for GA and PSO is noted as 87 ms and 63 ms respectively. Similarly, overshoot/undershoot time for GA is 1.71 ms and for PSO it is 1.96 ms. As noted the settling time for GA is 11.07 ms and for PSO it is 11.44 ms respectively. By observing these values from the Table 1 we can conclude that GA provides a better approach for solving the optimization problems. Therefore to summarize,

• Most literature suggests that STATCOM can function as a standalone device for integration into existing DFIG microgrids. However, detailed elaboration on coordinating a STATCOM with DFIG is limited. This highlights a research gap in establishing successful coordination control in a multi-STATCOM environment to ensure optimal performance and stability in a microgrid architecture.
• Much of the literature describes different communication protocols alongside distributed control architectures, emphasizing the functionality of the RBM-GA algorithm in hyperparameter tuning while focusing on static situations. However, there is minimal work guaranteeing the adaptability of algorithms under dynamic changing conditions in a microgrid architecture.
• Research on actual STATCOM-DFIG microgrids requires the implementation of real-time RBM-GA STATCOM control logic with FPGA and all interlocking mechanisms.

The remainder of the paper is organized as follows: Section 2 presents the problem formulation and mathematical modeling. In Section 3, the benchmarking model is introduced, where the GA-PI and PSO-PI controllers are described to enhance understanding of the model. Section 4 provides a detailed description of the experimental setup, followed by Section 5, which presents the analysis of the results and the conclusion is presented in Section 6.

## 2 Problem formulation

DFIG proves to be more robust compared to Permanent Magnet Synchronous Generator (PMSG) and Squirrel-Cage Induction Generator (SCIG) in terms of grid connectivity. It combines the advantages of both PMSG and SCIG, offering variable speed operation, efficiency, and simplicity at a lower cost. DFIG effectively addresses the controllability issues associated with SCIG, enabling both active and reactive power control. Additionally, it facilitates achieving power quality indicators such as voltage regulation and frequency control. Its ability to produce power at various wind velocities sets it apart from SCIG and PMSG. Hence, this research article focuses on DFIG over the other options

Induction generators typically operate under steady-state conditions with minimal slip and speed variations. When the slip approaches zero in such situations, the reactive power absorbed by the machine is relatively insignificant. However, as the load and power demand on the generator increase, both the slip and reactive power consumption of the motor also rise. Therefore, it becomes essential to adjust the stator voltage and flux for optimal performance and efficiency [22].

Regulating the stator voltage enables the induction generator to maintain the desired level of active power output while managing the reactive power requirements. By controlling the stator voltage, the magnetic field strength, which affects the generator's power delivery and response to load changes, can be managed effectively. Increasing the stator voltage compensates for higher load demands, preventing excessive slip and minimizing reactive power consumption [23], [24]. The stator voltage and Flux equation is presented at eq. (1). As observed the stator voltage is a function of stator flux and that of flux is a function of mutual inductance.

(1) $$\left\{ \begin{array}{c} V_{s}=R_{s}I_{s}+\frac{d\phi_{s}}{dt}\\ \phi_{s}=L_{s}I_{s}+L_{m}I_{r} \end{array}\right.$$

Similarly, maintaining control over the flux within the induction generator is crucial for stable operation under varying load conditions. Flux control techniques involve adjusting the magnetic field strength, which directly impacts the generator's torque and power output capabilities. Proper flux management allows the induction generator to adapt to load fluctuations, ensuring efficient operation while keeping slip and reactive power within acceptable limits. Similarly, the rotor flux as presented at eq. (2).

(2) $$\left\{ \begin{array}{c} V_{r}=R_{r}I_{r}+\frac{d\phi_{r}}{dt}-j\omega_{m}\phi_{r}\\ \phi_{r}=L_{r}I_{r}+L_{m}I_{s} \end{array}\right.$$

By using (1) and (2) the rotor and stator voltage equation becomes a complex conjugate function is presented at eq. (3).

(3) $$\left\{ \begin{array}{c} V_{r}=R_{r}I_{r}+sj\omega_{s}L_{\sigma r}+sj\omega_{s}L_{m}(I_{r}+I_{s})\\ V_{s}=R_{s}I_{s}+j\omega_{s}L_{\sigma r}I_{s}+j\omega_{s}L_{m}(I_{r}+I_{s}) \end{array}\right.$$

where “s” represents the slip of the system. Again by using (3) the stator active power and rotor active power is presented at eq. (4).

(4) $$\left\{ \begin{array}{c} P_{s}=\frac{3}{2}Re(V_{s}.I_{s})=\frac{3}{2}R_{s}{\left(I_{s}\right)}^{2}+\frac{3}{2}\omega_{s}L_{m}R_{s}(j(I_{r}.I_{s}))\\ Q_{s}=\frac{3}{2}Re(V_{r}.I_{r})=\frac{3}{2}R_{r}{\left(I_{r}\right)}^{2}+\frac{3}{2}s\omega_{s}L_{m}R_{s}(j(I_{s}.I_{r})) \end{array}\right.$$

Again the three phase line voltage is given by

(5) $$\left\{ \begin{array}{c} V_{ag}=I_{a}R_{a}+L\frac{dI_{a}}{dt}+V_{fa}\\ V_{bg}=I_{b}R_{b}+L\frac{dI_{b}}{dt}+V_{fb}\\ V_{cg}=I_{c}R_{c}+L\frac{I_{c}}{dt}+V_{fc} \end{array}\right.$$

Deriving the d-q component from eq. (5) it becomes

(6) $$\left\{ \begin{array}{c} V_{d}=I_{d}R+L\frac{dI_{d}}{dt}-L\omega I_{q}+V_{fd}\\ V_{q}=I_{q}R+L\frac{dI_{q}}{dt}+L\omega I_{d}+V_{fd} \end{array}\right.$$

The eq. (6) is known for its three distinct and conflicting characteristics. These characteristics encompass voltage compensation, a decoupling component, and a correction factor. In the context of the discussed research, the voltage correction factor has been effectively implemented using a STATCOM.

To begin with, eq. (6) is closely associated with voltage compensation, which involves the adjustment of voltage levels to maintain a desired value. Voltage compensation holds significant importance in electrical systems as it ensures stable and dependable operation, particularly when dealing with variations in load or disturbances in the grid.

Furthermore, eq. (6) incorporates a decoupling item. In the realm of power systems, decoupling refers to the process of isolating or separating interconnected parameters or variables. Its objective is to eliminate or minimize the mutual influence between these variables, thereby enabling superior control and regulation. The decoupling item present in equation (6) fulfills this purpose by facilitating independent control of specific variables within the system.

Now, the reference current control loop equation (ref. eq. (7)) becomes

(7) $$\left\{ \begin{array}{c} {I_{d}}^{⁎}=\frac{P^{⁎}V_{fd}+Q^{⁎}V_{fd}}{{V_{fd}}^{2}+{V_{fq}}^{2}}\\ {I_{q}}^{⁎}=\frac{P^{⁎}V_{fq}-Q^{⁎}V_{fd}}{{V_{fd}}^{2}+{V_{fq}}^{2}} \end{array}\right.$$

In order to achieve Unity power factor, ${I_{q}}^{⁎}=0$ and therefore

(8) $$\theta =\tanh\frac{V_{\beta }}{V_{\alpha }}$$

The magnitude of the power converter i.e. STATCOM Voltage can be proportional to DC Voltage and hence

(9) $$\left\{ \begin{array}{c} V_{d}=mV_{dc}cos(\theta )\\ V_{q}=mV_{dc}sin(\theta ) \end{array}\right.$$

and therefore by utilizing eq. (6), (8) and (9) the power balance equation becomes

(10) $$\left\{ \begin{array}{c} P=\frac{3}{2}(V_{d}I_{d}+V_{q}I_{q})\\ i_{dc}=\frac{3}{2}m(i_{d}cos(\theta )-i_{q}sin(\theta )) \end{array}\right.$$

Both zero dynamic technique and pole placement assignment system has been adopted along with GA-RBM to optimize the poles in an optimization theory. Here the control of STATCOM output as a function of DFIG control sequence has been used as the parameters like internal dynamics of a system that are not observable from its output. The state space equation for a linear system is

(11) $$\left\{ \begin{array}{c} x(t)=Ax(t)+Bu(t)\\ y(t)=Cx(t)+Du(t) \end{array}\right.$$

Now, representing eq. (10) adhering to eq. (11) the PI-controller gains are

(12) $$\left\{ \begin{array}{c} K_{d}(s)=K_{pi1}+\frac{K_{xxi}}{S}\\ K_{q}(s)=K_{pi2}+\frac{K_{xxj}}{S} \end{array}\right.$$

Here in eq. (12), $K_{pi1}$ and $K_{pi2}$ represents proportional and $K_{xxi}$ and $K_{xxj}$ represent integral gain. The state variables of current controller are defined as

(13) $$X_{3}(s)=\frac{K_{xxi}(I_{tdref}(s)-I_{d}(s))}{S}$$

and

(14) $$X_{4}(s)=\frac{K_{xxj}(I_{tqref}(s)-I_{q}(s))}{S}$$

Eq. (13) and eq. (14) do not directly impact the system's external behavior; however, they are important parameters for understanding the system's stability and robustness.

## 3 Bench-marking model

The coordinated control action between STATCOM and DFIG can be established either from the grid side based on grid side power quality or can be established from the source side based on environmental parameters such as wind speed. In both cases, the objective is to damp out the local oscillation in power quality during transient disturbances. The grid side controller (GSC) and source side controller (SSC) are connected back to create an independent control architecture. In order to investigate the robustness of the proposed controller two benchmarking model has been discussed and compared. Here the coupling capacitor voltage across the two converter models has been considered for STATCOM analysis. Therefore, the STATCOM output will act as a coordinated control output for GSC and SSC. The two bench-marking models are as follows.

### 3.1 Case-1:- GA optimized PI-controller

The Genetic Algorithm (GA) is a type of global search algorithm that draws inspiration from the mechanics of nature, particularly concepts like natural selection and the survival of the fittest, as well as principles from genetics. Its primary purpose is to optimize complex and challenging parameters associated with controllers in order to solve traditional optimization problems effectively. The strength of the GA lies in its capacity to harness historical information obtained from previous solutions. By leveraging this knowledge, the algorithm aims to enhance the performance of future solution structures. To facilitate this process, GA maintains a population of individuals that represent potential solutions to the problem at hand. Each individual within the population is assessed and assigned a fitness value based on their suitability with respect to the objective function.

The ultimate objective in GA is to minimize the objective function, which in turn improves the system's response. This is achieved by considering various parameters, including but not limited to the rise time, settling time, and peak overshoots. Moreover, the problem itself imposes certain constraints, such as the need for a lead-lag configuration, the inclusion of a washout filter, and the optimization of parameters for the proportional-integral (PI) controller within the damping controller. As a result, the process of parameter optimization can be formulated as an optimization problem. The goal is to determine the optimal set of parameters that not only minimize the objective function but also adhere to the specified constraints.

The main challenge in designing coordinated parameters with another controller is selecting a suitable input signal. This input signal plays a crucial role in providing effective control actions when disturbances arise in the system. Several factors are taken into consideration when choosing the input signal, such as line reactive power, active power, bus magnitude of the current, and voltage. In the context of a coordinated proportional-integral (PI) controller, the input signal selected is the rotor speed deviation of the DFIG. This specific signal captures the desired oscillations required for dampening inherent and regular oscillations, particularly related to coupling voltage. By utilizing the rotor speed deviation as an input signal, the coordinated PI controller aims to address chronic oscillations during dynamic conditions.

An Objective Function, denoted as “f,” is defined based on the rotor speed deviation to achieve this objective. The purpose of this function is to minimize chronic oscillations and enhance the system's capability. The evaluation of the objective function takes into account various performance metrics, including settling time, peak overshoot, and rise time, as well as the presence of both un-damped and damped oscillations. By optimizing these parameters, the objective function strives to improve the overall system response and stability.

**Figure 1:**
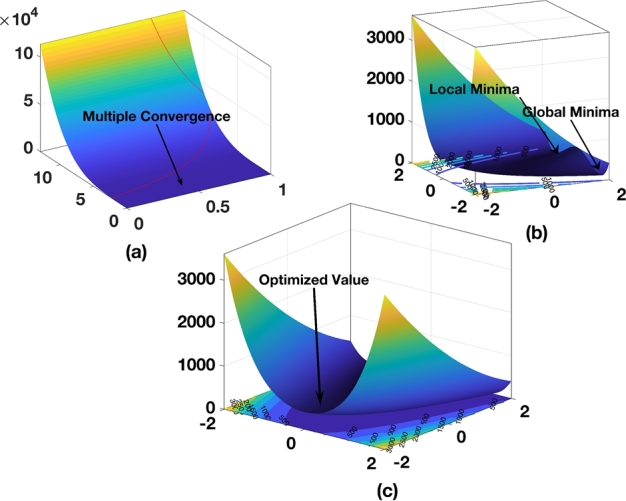
Optimization surface analysis a) Population size P=110 b) Population size P=60 c) Population size P=30.

Fig. 1 represents the optimization surface analysis for three different population sizes. In Fig. 1(a), the analysis is shown for a population of P = 110. Multiple convergence points can be observed for the three batches as a whole. This indicates that the GA value is 4.341 and the P value is $5.581\times 10^{-4}$. Due to the presence of multiple convergences, the algorithm does not provide a specific solution. To further extend the analysis, a second batch with a population size of 60 is considered. Fig. 1(b) represents the surface optimization for this population size, where both local and global minima are present. Therefore, in the 3-D plane, there are four minima in addition to the global minimum. Consequently, the algorithm becomes trapped in one of the minima instead of reaching the global maximum. Subsequently, in Fig. 1(C), the analysis was performed for a population size of P = 30, where a single solution exists on the surface, with a value of 0.378. For further analysis, a maximum batch size of a population of 30 is considered.

**Figure 2:**
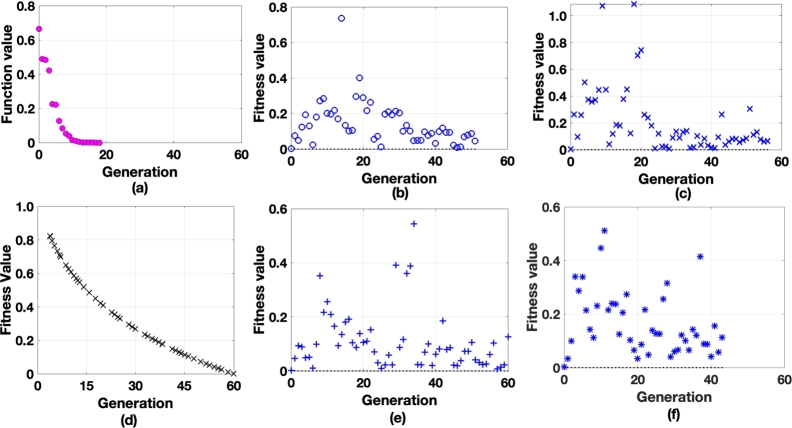
Fitness function evaluation as a function value vs. generation for different elite levels a) c=0.17 b) c=0.23 c) c=0.27 d) c=0.33 e) c= 0.36 f) c= 0.41.

Fig. 2 represents the analysis of functional values in relation to generation data. Six different analyses were conducted with different elite individuals. Fig. 2(f) shows that the system has random variables ranging from 0.003 (minimum) to 0.52 (maximum), indicating incomplete solutions for the corresponding elites. Likewise, Fig. 2(a) illustrates the analysis of the elite individual with c=0.17. The system exhibits a rectangular hyperbola solution during the optimization process. To assess the algorithm's effectiveness, a 6-batch analysis was performed using three different population sizes: P=10, 20, and 30. From Table 2, it can be observed that the minimum standard deviation for batch-1 is 0.068, while the maximum standard deviation is 0.929. Similarly, for batch 2, the standard deviation ranges from 0.735 to 1.015, and for batch 3, it ranges from 0.749 to 1.106.

**Table 2:** Batch analysis of Chromosome configuration in the GA-Optimization process.

Sr. No.	Batch	No. of Candidate	Mean GA	STD-GA	P-Value
01	B-1	10	4.273	0.668	5.581 E-4
	B-2	20	4.409	0.735	5.805 E-4
	B-3	30	4.582	0.749	6.018 E-4

02	B-1	10	4.478	0.681	5.810 E-4
	B-2	20	4.593	0.744	6.025 E-4
	B-3	30	4.734	0.811	6.285 E-4

03	B-1	10	5.725	0.859	7.479 E-4
	B-2	20	5.908	0.984	7.779 E-4
	B-3	30	6.139	1.003	8.064 E-4

04	B-1	10	6.000	0.912	7.813 E-4
	B-2	20	6.154	0.996	8.074 E-4
	B-3	30	6.343	1.086	8.381 E-4

05	B-1	10	5.831	0.911	7.617 E-4
	B-2	20	6.017	1.003	7.922 E-4
	B-3	30	6.253	1.022	8.213 E-4

06	B-1	10	6.111	0.929	7.957 E-4
	B-2	20	6.268	1.015	8.223 E-4
	B-3	30	6.460	1.106	8.536 E-4

Based on the batch analysis mentioned above, the algorithm has been standardized for a batch size of P=30, with an elite individual level of c=0.377. Fig. 2(b) and Fig. 2(e) represent the scattered analysis of fitness value Vs generation at an elite level of c=0.23 and c=0.36 respectively. As observed most of the data were accumulated between 10 to 40 generations. Similarly, in Fig. 2(c) and Fig. 2(d) the optimization process has been presented. The same was and analyzed through experimental analysis and compared with the proposed model as a benchmark.

### 3.2 Case-2:- PSO optimized PI-controller

**Figure 3:**
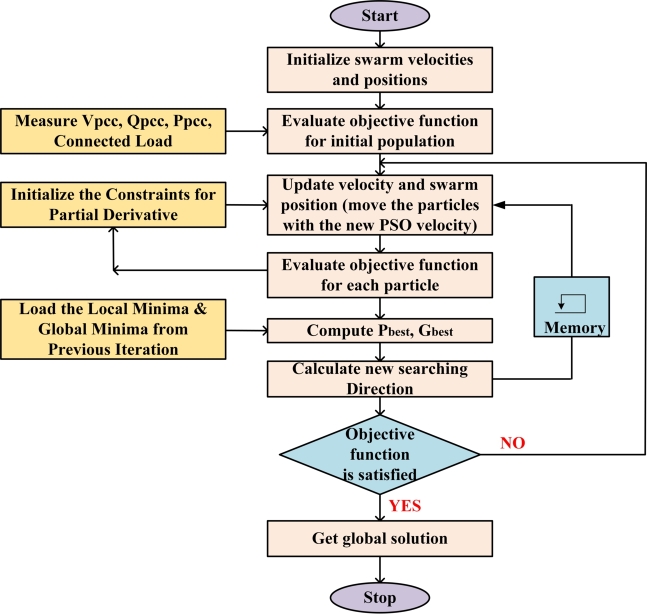
Flowchart of Particle Swarm Optimization (PSO).

An algorithm for stochastic population-based optimization is known as particle swarm optimization (PSO). PSO maintains two populations: a population of the particle's best locations (i.e., $G_{best}$) and a population of the particle's current positions (i.e., $P_{best}$). In the search space, the former is considered as a candidate solution, while the latter is employed to guide the former's update. Each particle in the PSO system has two characteristics, a velocity vector V and a position vector X, and moves through the search space at a velocity that is dynamically changed in response to the experiences of both the particles and their companion. The PSO algorithm's flowchart is shown in Fig. 3. The following mathematical formula is used to update the particle's position and velocity [1]:

(15) $$V_{id}(t+1)=\omega ⁎V_{id}(t)+c_{1}⁎r_{1}⁎[P_{id}(t)-x_{id}(t)]+c_{2}⁎r_{2}⁎[P_{gd}-x_{id}(t)]$$

(16) $$x_{id}(t+1)=x_{id}(t)+v_{id}(t+1)$$

**Figure 4:**
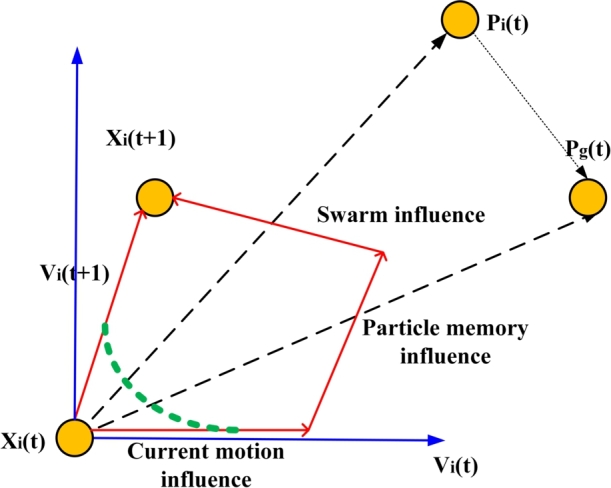
Graphical representation of the particle evolution.

Where the acceleration coefficients $c_{1}$ and $c_{2}$ represent the weight of the stochastic acceleration factors that push each particle in the direction of its $p_{best}$ and $g_{best}$ positions respectively. Two random numbers that are uniformly distributed between 0 and 1 are represented by the symbols $r_{1}$ and $r_{2}$. It resembles the temperature parameter in the simulated annealing (SA) in terms of its characteristics. The inertia weight *ω* is used to balance the global and local searches. A large inertia weight typically makes global exploration easier while a lower inertia weight typically makes local exploitation easier. A1−dimensional vector can be used to represent the $i^{th}$ particle's position as $X_{i}=[x_{i1},x_{i2},\ldots x_{ij},\ldots x_{iD}]$ where $x_{ij}=[x_{min},x_{max}]$ $j_{i}^{th}$ position of the $i_{th}$ particle position and the corresponding velocity is $V_{i}=[v_{i1},v_{i2},\ldots v_{ij},\ldots v_{iD}]$ where $v_{ij}=[v_{min},v_{max}]$. The position of best fitness value is denoted by $P_{i}=[p_{i1},p_{i2},\ldots p_{ij},\ldots p_{iD}]$ while $g_{best}$ recorded so far is $P_{g}=[p_{g1},p_{g2},\ldots p_{gj},\ldots p_{gD}]$. The particle evolution is presented graphically in Fig. 4.

**Figure 5:**
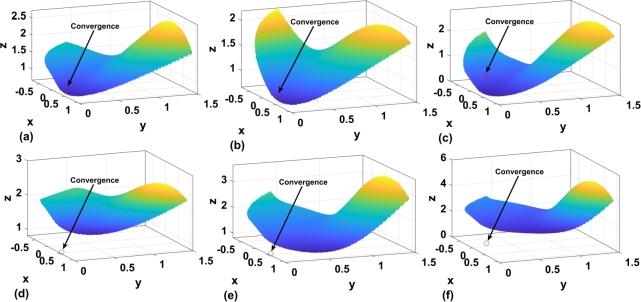
Optimization surface analysis at different velocity (a) v1=0.33 (b) v=0.31 (c) v=0.27 (d) v=0.26 (e) v=0.21 (f) v=0.18.

Fig. 5 represents the optimization surface analysis with a heat map for convergence. In three situations, from Fig. 5 (d) to Fig. 5 (f) the intersection is outside boundary condition for velocity levels of 0.26,0.21, and 0.18 respectively. In Fig. 5(c), the convergence shifted towards the left hand side of the plane making it unsuitable for convergence analysis. Similarly, for Fig. 5 (b), the marginality of the boundary is greater as compared to other velocities represented through yellow and brown maps. However, for Fig. 5 (a), the convergence happens at 0.314. Similarly, for Fig. 5(e), the intersection occurs at the margin and again the gain in this case is not dynamic with respect to variation in reactive power demand. Therefore, PSO with a swarm velocity level of 0.33 has been considered for further analysis.

**Figure 6:**
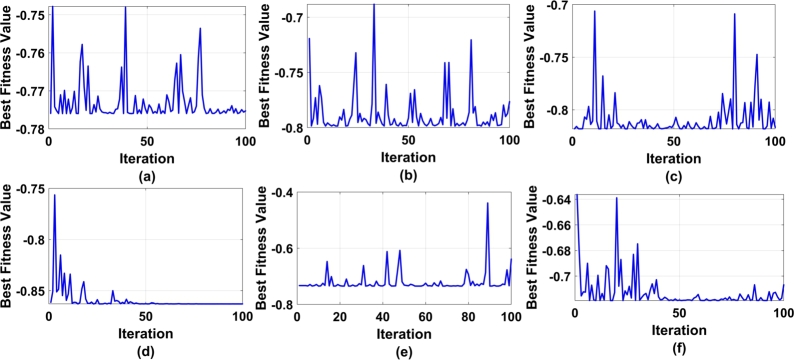
PSO fitness evaluation a) m1=0.77 b) m2=0.64 c) m3=0.53 d) m4=0.39 e) m5=0.27 f) m6=0.21.

Fig. 6 shows the PSO fitness against no. of iteration. Here for optimization, 100 iterations have been considered with different lambda values in the range of [0.21 0.77]. Fig. 6 (f) shows the best fitness values (Fig. 6 (a) - Fig. 6 (e)). The maximum standard deviation is 0.041. Therefore, a lambda value of 0.46 has been considered for further validation of the algorithm under the experimental section.

## 4 Simulation model

**Figure 7:**
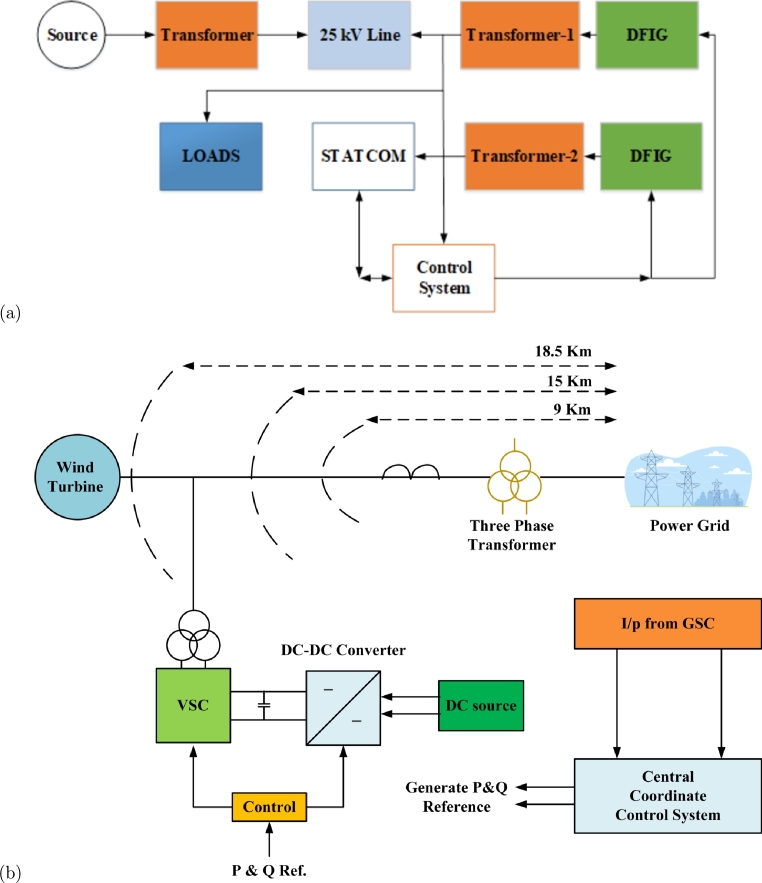
(a) Block diagram of Experimental setup in MATLAB (b) Block Diagram of Wind-STATCOM coordinate control system.

The experimental setup was employed in the proposed study to investigate the coordinated control action between DFIG and STATCOM. The configuration, as illustrated in Fig. 7(a), consisted of a swing bus, a transformer, and 25 Km transmission lines arranged in a left-to-right sequence. In addition, another wind power plant, connected through TFR-1, consisting of three turbines, each with a 26KVA capacity, was situated at a distance of 23 km from the central power center. Similarly, the block diagram of the wind-STATCOM coordinate control system is presented in Fig. 7(b). The DFIG's particulars are detailed in Table 3, and its wind turbine control structure includes two converters, namely, the grid side converter control system and the rotor side converter control system. The grid side converter's main function is to control and establish the capacitor voltage, which is also employed to align the d-axis and q-axis current.

**Table 3:** Detail parameter of Doubly fed Induction Generator.

Sr. No.	Parameter	Rating	Unit
1	Nominal Line to Line RMS Voltage	400	V
2	Nominal Frequency	50	Hz
3	Nominal Full load Line Current	38.5	A
4	Nominal Full Load Mechanical Torque	82	N-m
5	Synchronous Speed	3000	RPM
6	Nominal Mechanical Speed	2900	RPM
7	Starting Current to Nominal Current Ratio	7.3	
8	Starting Torque to Full Load torque ratio	2.48	
9	Breakdown torque to full load torque ratio	2.61	
10	Nominal Power Factor	88	pf
11	Stator Resistance	0.3067	ohm
12	Stator Inductance	0.003747	H
13	Mutual Inductance	0.05157	H
14	Generator Rating	47	KVA
15	Pole Pairs	1	

Here the q-axis component of the control structure is set to zero to generate a unity power factor across the output. Again the reference voltage, i.e., Vdc in is also set to a higher voltage like 500 V for a grid side voltage level of 400 V ac. Here in this model, two PI-controllers have been used instead of one PI-controller to increase the controller's performance. The detail parameter for the grid side converter is shown in Table 4. It can be seen that here the actual DC voltage is around 528 V, and that of the reference modulation index of 0.92. The controller's optimum result can be obtained by seeing the Modulation index between 0.9 to 1.0; in this paper, it has been considered as 0.92. The detailed parameter for designing the rotor side converter using a PI controller is shown in Table 5.

**Table 4:** Detail parameters of Grid Side Converter.

Sr. No.	Parameter	Rating	Unit
1	Vdc	1150	V
2	Vdc_Actual	528	V
3	Id_ref	-0.8	A
4	Iq_ref	0	A
5	Id_act	0.788	A
6	Iq_act	-0.23	A
7	Vd_actual	0.27	V
8	Vq_act	0.104	V
9	M-Index	0.92	NA

**Table 5:** Detail parameter of Rotor Side Converter.

Sl. No.	Parameter	Rating	Unit
1	Inductive Load	22	KVA
2	Current Rating	37.8	A
3	Series Inductance	800	mH
4	Coupling Inductance	648.7	mH
5	Capacitor Capacitance	10000	mF
6	Modulation Index	0.92	NA
7	AC voltage Reference Point	1.015	P.U
8	DC Voltage SetPoint	2400	V
9	Kp-Vac	0.55	NA
10	Ki-Vac	2500	NA
11	Kp-Vdc	0.001	NA
12	Ki-Vdc	0.15	NA
13	RC Load	7.25	KVAR

**Figure 8:**
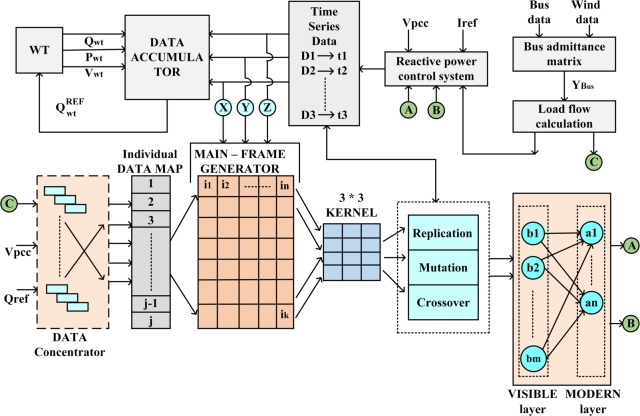
Schematic Diagram implementing Optimized Restricted Boltzmann Machine Algorithm in DFIG Microgrid architecture.

Fig. 8, represents the schematic diagram for implementing the Optimized Restricted Boltzmann Machine Algorithm in DFIG Microgrid architecture. As observed, the data concentrator upon receiving the three input parameter such as load-flow calculation Index, $V_{pcc}$ and $Q_{ref.}$, map with individual data map to create a frame generator. The frame generator, using Kernel will initiate the GA based RBM-1 model to prepare control signal for reactive power control system model. The reactive power control system generates time series data, which will be fed to data accumulator to generate Ref. reactive power for wind turbine.

## 5 Result analysis

**Figure 9:**
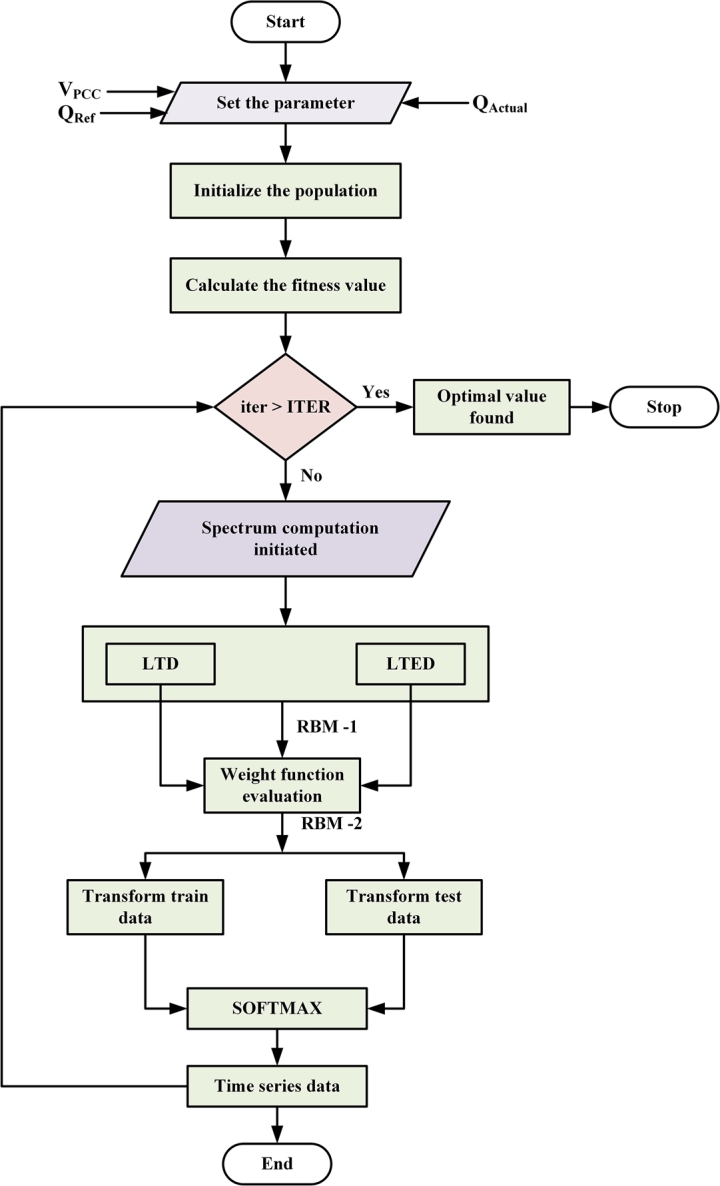
Flowchart implementing GA Optimized Restricted Boltzmann Machine Algorithm in Parameter tuning.

To start with the flow chart Fig. 9 represents the basic process diagram of GA enabled restricted Boltzmann algorithm. During the initial stage, 3-parameters were accumulated such as $V_{pcc},Q_{ref}andQ_{act}$. A population size of 500 was set in the program to start the GA optimization. Fitness value was evaluated based on the data set, with an emphasize to minimize the RMSE error after each epoch operation. However, upon failing of the iterations a spectrum of data frame was created to segregate the input data in terms of the data labeled training data (LTD) and labeled testing data (LTED). RBM-1 model component was initiated for evaluating the wt. function followed by initiation of RBM-2 model, where transformation matrix has been applied to train and test data respectively. Softmax algorithm has been used to replace a time series data, which will determine the proportional and integral gain of the system. Based on the flowchart (ref. Fig. 9) description, the STATCOM wind microgrid model has been evaluated with three different controller tuning algorithm.

**Figure 10:**
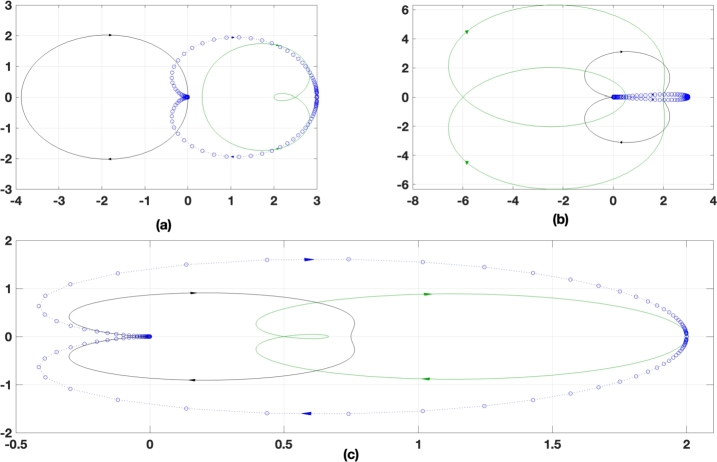
Grid Side Controller Transfer function analysis a) Firefly-RBM optimized PI controller b) PSO-RBM optimized PI Controller c) GA-RBM optimized controller.

Table 6 represents the comparative analysis of different gains at different time constants with different algorithms. The system has been evaluated with five different time constants in between [0.0- 0.1]. With the increase in time constant, the system performance becomes more sluggish and that of frequency response also becomes towards the unstable region. A detailed analysis for each algorithm has also been analyzed in the frequency domain using the Nyquist plot. Fig. 10 shows the Nyquist analysis of PI controller for different algorithms. From Fig. 10(a), the Nyquist plot analysis shows that with Firefly-RBM algorithm it is possible to add 11.37% of gain to the transfer function, whereas with GA-RBM it is able to add 29.07% of gain to the transfer function under maximum reactive power demand, occurring due to sudden load thrown off or sudden loading condition. Similarly with Fig. 10 (b), and in Fig. 10(c) the allowed gain percentage is 18.76% and 21.04% respectively. Further analysis in this paper has been carried out with GA-RBM optimized PI controller for synchronizing the STATCOM with microgrid in coordination with the Wind turbine system for active and reactive power compensation.

**Table 6:** Comparative analysis of gain at different time constant and algorithm.

Type of Algorithm	Gain Parameter	Time Constant				
		0.0	0.25	0.50	0.75	1.0
GA	Kp	0.348	0.342	0.308	0.237	0.170
	Ki	0.541	0.535	0.482	0.371	0.265

PSO	Kp	0.323	0.319	0.288	0.221	0.158
	Ki	0.492	0.487	0.438	0.337	0.241

FPA	Kp	0.317	0.328	0.308	0.245	0.169
	Ki	0.471	0.453	0.451	0.322	0.232

GA-RBM	Kp	0.301	0.298	0.268	0.206	0.147
	Ki	0.447	0.442	0.398	0.306	0.219

**Figure 11:**
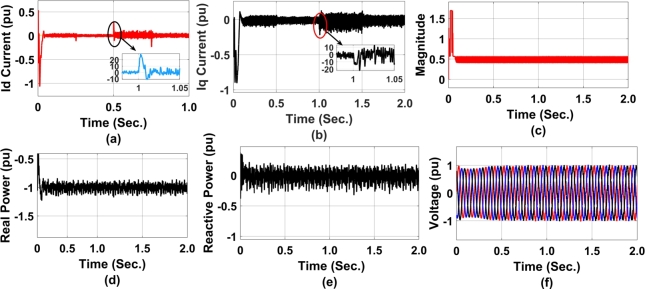
GA-enabled coordinated controller performance from STATCOM to DFIG a) direct axis current reference for RSC b) direct axis current reference for RSC c)Duty cycle of converter d) Real power exchanged between STATCOM and DFIG e) Reactive Power exchanged between STATCOM and DFIG f) Voltage at PCC.

Fig. 11 represents GA-enabled coordinated controller performance between STATCOM and DFIG. The direct and quadrature axis current calculated using GA is presented in Fig. 11 (a) and Fig. 11 (b). It is observed that at time t = 0.5 sec the system has undergone a transient disturbance for 1 cycle of operation. During this cycle a current glitch of 20% higher than the normal operating condition has been observed. Fig. 11(c), represents the duty cycle of the converter and that of Fig. 11 (d) represents the real power exchanged between statcom and grid. Due to the change in direct access current a significant amount of current change in the quadrature axis has also been noticed. Due to the presence of transient disturbances, the duty cycle of the converter is maintained at half magnitude as compared to full load. The real power is drawn out from the grid to the STATCOM at 1 PU with some noise. The noise is again due to the presence of the DFIG rotor side controller. The consequent variation in the reactive power due to STATCOM action, the reactive power is maintained at 0PU with negative variations in the form of noise, Fig. 11(e). The three-phase voltage at PCC is represented in Fig. 11(f). As observed, a voltage swell has occurred at 0.5 sec, to meet the reactive power demand due to the interaction of DFIG and STATCOM.

**Figure 12:**
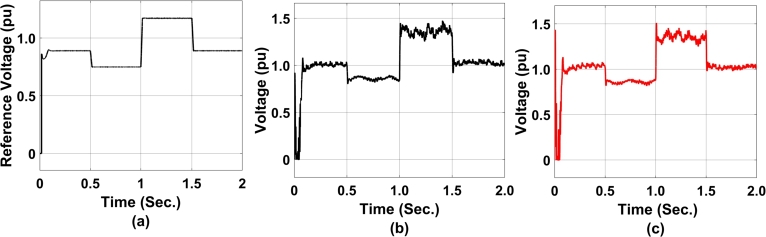
GA-enabled coordinated controller performance from STATCOM to DFIG a) DC-ref. voltage b)positive terminal voltage at STATCOM c)negative terminal voltage at STATCOM.

Fig. 12 represents GA-enabled controller performance along with STATCOM converter terminal voltage performance. At Fig. 12(a), the reference voltage is maintained at 0.8PU before undergoing a perturb change in reactive power demand at the grid. Again it is worthwhile to mention here that both STATCOM and DFIG are in an interlocked manner from the grid side controller point of view. This also has an impact on reactive power demand and hence on voltage as a function of rotor side controller gain. Similarly, Fig. 12(b) and Fig. 12(c) represents the positive and negative terminal voltage of the STATCOM converter. In contradiction to the reference voltage, the positive and negative voltage is maintained at 1PU before taking a perturb at 0.5 sec. This is a transient disturbance between 1 sec to 1.5 sec, because of active power filter action to reduce the dynamic harmonics and wind gust disturbances transferred from the rotor side controller to the grid side controller.

**Figure 13:**
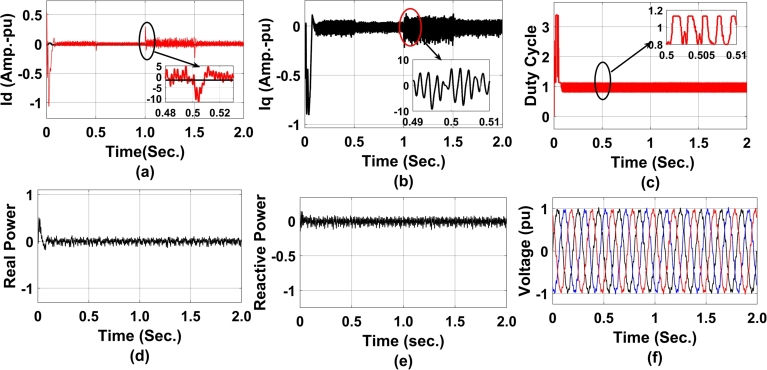
PSO-enabled coordinated controller performance from STATCOM to DFIG a) direct axis current reference for RSC b) direct axis current reference for RSC c)Duty cycle of converter d) Real power exchanged between STATCOM and DFIG e) Reactive Power exchanged between STATCOM and DFIG f) Voltage at PCC.

Fig. 13, represents the PSO-enabled coordinated controller performance from STATCOM to DFIG where Fig. 13(a) represents the direct axis current reference for RSC, where it can be observed that the system has undergone a transient disturbance of slope 2.23% before settling down to reference level. Similarly, Fig. 13 (b) represents the direct axis current reference for RSC, and Fig. 13 (c) shows the Duty cycle of the converter. Similarly, Fig. 13(d) and Fig. 13(e) shows the Real power and Reactive Power exchanged between STATCOM and DFIG and that of Fig. 13(f) shows the Voltage at PCC.

**Figure 14:**
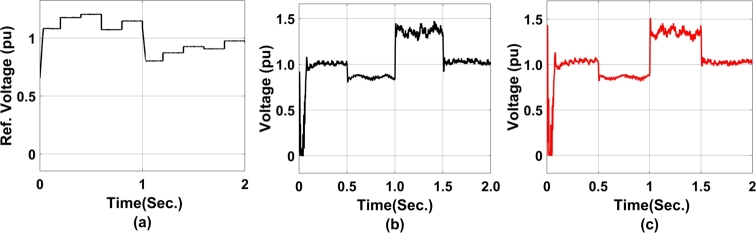
PSO-enabled coordinated controller performance from STATCOM to DFIG a) DC-ref. voltage b)positive terminal voltage at STATCOM c)negative terminal voltage at STATCOM.

Fig. 14, represents the PSO-enabled coordinated controller performance from STATCOM to DFIG. The step change in reference (Fig. 14(a)) represents the swarm control decision of voltage. Fig. 14(b) and Fig. 14 (c) represents the positive and negative terminal voltage at STATCOM respectively. As observed there is no spike in positive terminal voltage however at negative terminal voltage there is a steep spike at 1 sec. with respect to the timeline.

GA-RBM utilizes a combination of RBM and GA algorithms to construct a model that learns from Phase Locked Loop (PLL) captured data and optimizes RBM weights for improved performance. The GA algorithm chooses the most adept individuals from the population, reproduces them, and generates a new generation of individuals with enhanced fitness levels, thereby facilitating RBM weight optimization.

**Figure 15:**
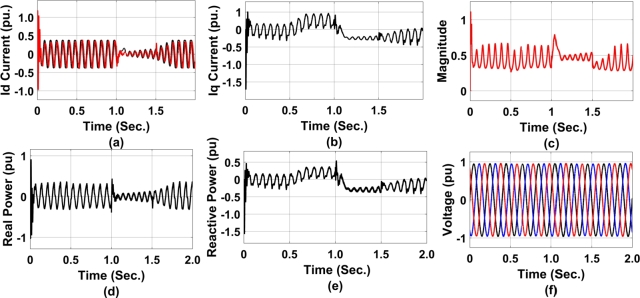
GA-RBN-enabled coordinated controller performance from STATCOM to DFIG a) direct axis current reference for RSC b) direct axis current reference for RSC c)Duty cycle of converter d) Real power exchanged between STATCOM and DFIG e) Reactive Power exchanged between STATCOM and DFIG f) Voltage at PCC.

The direct and quadrature axis current for the entire duration of the simulation is presented in Fig. 15 (a) and (b) respectively. As observed the quadrature axis current under fault condition is a little bit swell indicating the generation of reactive power to support the change in voltage as desired. The duty cycle of the converter is presented in Fig. 15 (c). The real and reactive power exchanged between SCIG and Microgrid is presented in Fig. 15(d). Initially, the system starts with stable operating conditions in terms of power delivery. At about 1.0 sec., the SCIG reduces its speed as a result there is a decrease in the real power delivery into the grid. This also results in a decrease in reactive power support (Fig. 15(e)) to the grid at PCC. At about 1.0 sec., upon the loss of speed, the system has achieved a sag in the real power delivery for 0.5 sec. and after that, the system got again stable, leading to stable reactive power support to the grid. Similarly, the uniformity in the voltage is presented in the Fig. 15(f).

**Figure 16:**
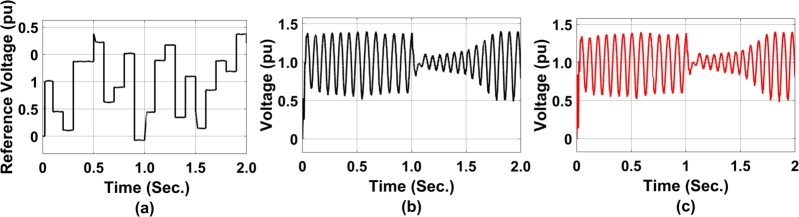
GA-RBN-enabled coordinated controller performance from STATCOM to DFIG a) DC-ref. voltage b)positive terminal voltage at STATCOM c)negative terminal voltage at STATCOM.

Fig. 16 represents the performance analysis of GA-RBN enabled coordinated control between STATCOM and DFIG. As observed in Fig. 16(a), the reference voltage has fluctuated between 0 to 0.48 pu. This means that during the actual condition of reference voltage evaluation, a maximum deviation of 0.48 pu of voltage will occur. Similarly, Fig. 16(b) represents, positive terminal voltage of STATCOM and that of negative component is presented in Fig. 16(c).

Table 7, represents the Power Quality variation analysis with STATCOM-Wind-Microgrid system under GA-RBN optimized control architecture. The different power quality parameters such as voltage swell, and sag for short-duration variations have not been carried out in this research article. The waveform distortion analysis parameters are also under the limit of IEEE reference standards. The harmonic content is about 8.27%, which is a little bit higher from STATCOM side, this is because of the use of a 9-level converter for the power conversion system.

**Table 7:** Power Quality variation analysis with STATCOM-Wind-Microgrid system under GA-RBN optimized control architecture.

Type of Variations	Power Quality Parameters	IEEE Standard		Actual Change in Voltage Magnitude
		Time Constant	Voltage Magnitude	
Short Duration Variations	Sag	3 Sec. to 1 Min	0.1 to 0.9 pu	3.7 sec./0.21 pu
	Swell	3 Sec. to 1 Min	1.1 to 1.2 pu	3.03 sec./1.1 pu
	Interuption	3 Sec. to 1 Min	<0.1 pu	NA

Long Duration Variations	Interruption	>1 Min.	0.0 pu	NA
	Undervoltage	>1 Min.	0.8 to 0.9 pu	2.27 Min./0.83 pu
	Overvoltage	>1 Min.	1.1 to 1.2 pu	1.3 Min./1.1 pu

Wave form Distortion	DC Offset	-	0 to 0.1%	0.03%
	Harmonics	-	0 to 20%	8.27%
	Interharmonics	-	0 to 2%	0.48%
	Notching	-	-	-
	Noise	-	0 to 1%	0.09%

Table 8 represents a Comparative Analysis of Confidence Intervals among different algorithms. As observed the GA-RBN has the best confidence interval of 91.88% as compared to PSO and GA taken for 95% of data consideration. Similarly, for a confidence interval level of 98%, the GA-RBN source had 92.71% of accuracy as compared to GA and PSO. A detailed comparative analysis for algorithms in terms of tracking, decision, and Command time against % of load change has been presented in Table 9. It is observed that for all the loading conditions the system has a better performance as compared to another algorithm in terms of data tracking and decision time.

**Table 8:** Comparative Analysis of Confidence Intervals among different Algorithms.

Sr. No.	Algorithm	Median- Cost	Confidence Interval (92%)	Confidence Interval (95%)	Confidence Interval (98%)
1	GA	0.41	88.26	90.07	90.88
2	PSO	0.33	89.14	90.97	91.79
3	GA-RBN	0.27	90.03	91.88	92.71

**Table 9:** Comparative Analysis of algorithms in terms of tracking, decision, and Command time against % of load change.

Sr. No.	Algorithm	% of Load Change	Tracking Time	Decision Time	Command Time
1	GA	15	0.082	0.067	0.043
2		30	0.084	0.069	0.044
3		45	0.087	0.071	0.046
4		60	0.090	0.073	0.047
5		75	0.092	0.075	0.048

6	PSO	15	0.080	0.066	0.042
7		30	0.083	0.068	0.043
8		45	0.085	0.070	0.045
9		60	0.088	0.072	0.046
10		75	0.090	0.074	0.047

11	GA-RBN	15	0.079	0.064	0.041
12		30	0.080	0.066	0.042
13		45	0.082	0.067	0.043
14		60	0.084	0.068	0.044
15		75	0.085	0.070	0.045

## 6 Conclusion

A detailed analysis of the Proposed GA-RBM for STATCOM in a microgrid has been presented in this research article. The RBM uses a series of k Gibbs sampling steps to estimate the gradient of the log-likelihood function, instead of just a single Gibbs sampling step used in traditional contrastive divergence. This leads to faster convergence and better performance, especially for microgrid range of data sets. The classical STATCOM controller has a limited ability to control complex and dynamic power systems, particularly in regulating voltage and reactive power in systems with high levels of variability and uncertainty. Optimizing the parameters of the STATCOM controller using GA results in enhanced performance in regulating voltage and reactive power in complex and dynamic power systems. This optimization enables better control of the system and faster response times to changes in the system, leading to improved overall performance.

## Funding

No funding was supported for this research work.

## CRediT authorship contribution statement

**Rajeswari Bhol:** Writing – original draft, Visualization, Validation, Software, Methodology, Investigation, Data curation, Conceptualization. **Sarat Chandra Swain:** Writing – review & editing, Writing – original draft, Visualization, Supervision, Software, Resources, Methodology, Investigation, Data curation, Conceptualization. **Ritesh Dash:** Writing – review & editing, Visualization, Validation, Resources, Methodology, Formal analysis, Data curation, Conceptualization. **K. Jyotheeswara Reddy:** Writing – review & editing, Writing – original draft, Visualization, Resources, Methodology, Data curation. **C. Dhanamjayulu:** Writing – review & editing, Visualization, Validation, Supervision, Software, Project administration, Investigation, Funding acquisition, Data curation, Conceptualization. **Hossam Kotb:** Writing – review & editing, Writing – original draft, Validation, Software, Resources, Formal analysis, Data curation, Conceptualization. **Ahmed Emara:** Writing – review & editing, Writing – original draft, Visualization, Validation, Data curation, Conceptualization.

## Declaration of Competing Interest

The authors declare that they have no known competing financial interests or personal relationships that could have appeared to influence the work reported in this paper.

## Data Availability

The datasets used and/or analyzed during the current study are available from the corresponding author on reasonable request.
